# Therapeutic Potential of Circular RNAs as Targets for Cancer Treatment

**DOI:** 10.34172/apb.025.45330

**Published:** 2025-07-15

**Authors:** Milad Asadi, Sanam Sadeghi-Mohammadi, Najibeh Shekari, Venus Zafari, Zahra Soleimani, Ufuk Mert, Maryam Seyyedi, Ayse Caner, Habib Zarredar

**Affiliations:** ^1^Department of Basic Oncology, Institute of Health Sciences, Ege University, Izmir, Turkey; ^2^ATMP Department, Breast Cancer Research Center, Motamed Cancer Institute, ACECR, Tehran, Iran; ^3^Department of Immunology, Faculty of Medicine, Tabriz University of Medical Sciences, Tabriz, Iran; ^4^Rahat Breath and Sleep Research Center, Tabriz University of Medical Sciences, Tabriz, Iran; ^5^Atatürk Health Care Vocational School, Ege University, Izmir, Turkey; ^6^Tuberculosis and Lung Diseases Research Center, Tabriz University of Medical Sciences, Tabriz, Iran; ^7^Department of Parasitology, Faculty of Medicine, Ege University, Izmir, Turkey

**Keywords:** Circular RNA, Biogenesis, Diagnosis, Prognosis, Cancer

## Abstract

Circular RNAs (circRNAs) are a novel class of non-coding RNAs primarily generated through a back-splicing processes. These molecules exhibit extensive expression across various tissues, indicating their significant role in numerous biological processes, particularly in complex diseases such as cancer. Based on their origin, structure, and biogenesis, circular RNAs are categorized into exonic circRNAs (ecirc-RNAs), circular intronic RNAs (ci-RNAs), or exonic-intronic circRNAs (EIci-RNAs). Due to their covalently closed-loop configuration, it is necessary to develop specialized techniques to study them. CircRNAs are known to function as protein and microRNA sponges, regulate transcription, interact with RNA-binding proteins (RBPs), and, in rare cases, serve as templates for translation. In this review, we provide an overview of circRNA features, biogenesis, and functions. In addition, we summarize molecular methods for studying them and explain their significant roles in malignancies.

## Introduction

 Non-coding RNAs (ncRNAs) constitute the predominant class of transcribed RNAs in eukaryotic cells, and more than 90 % of the entire RNA expression is related to these types of RNAs. These molecules are broadly classified based on length into two major categories: small ncRNAs (sncRNAs, < 200 nt) and long ncRNAs (lncRNAs, > 200 nt).^1,2^ MicroRNAs (miRNAs), small nucleolar RNAs (snoRNAs), small nuclear RNAs (snRNAs), PIWI-interacting RNA (piRNAs), and small interfering RNAs (siRNAs),^3^ are the prominent members of sncRNAs.^4-6^ Conversely, lncRNAs include subtypes such as long intergenic ncRNAs, intronic ncRNAs, macroRNAs, sense ncRNAs, antisense RNAs, and circular RNAs (circRNAs).^7-9^ In eukaryotic systems, mRNA precursors (pre-mRNAs) commonly contain intronic sequences that are removed via canonical splicing to form mature, linear transcripts.^10^ However, under certain conditions, these processes can make an entirely different kind of RNA from the same precursor RNA. Initially described approximately 30 years ago, it was discovered that if during non-canonical splicing, specifically back-splicing, an upstream splice acceptor joins a downstream splice donor, circRNAs generating.^11,12^

 This kind of ncRNA contains covalently locked non-stop loop constructions (D-loop) without terminal 5’ caps and 3’ poly-A tails.^12,13^ It is theoretically possible for all internal exons of genes, excluding the first and last, to give rise to circRNAs. Although back-splicing is considered a relatively rare event, there are more than 200,000 exons in the human genome, and in contrast to the low occurrence of back-splicing, 1,000 unique circRNAs can be found in any given cell type.^14^ Despite their generally low expression levels, circRNAs exhibit resistance to exonuclease-mediated degradation due to their circular structure and have been implicated in several regulatory roles.^15^ These include modulation of parental gene expression, alternative splicing or translation, acting as miRNA or RNA-binding protein (RBP) sponges, translation into peptides/ proteins (only a few circRNAs), and the generation of some pseudogenes.^16^

 An increasing number of studies have revealed the aberrant expression of circRNAs in various pathological conditions, including cancers, neurological disorders, and cardiovascular diseases. In oncology, circRNAs can act as either oncogenes or tumor suppressors, depending on their targets and interactions.^17^ For instance, circHIPK3 promotes colorectal cancer progression by sponging multiple tumor-suppressive miRNAs,^18^ while circMTO1 suppresses hepatocellular carcinoma (HCC) via inhibition of the oncogenic miR-9. The stability and specific expression patterns of circRNAs in different tissues make them promising candidates for non-invasive diagnostic and prognostic biomarkers, as well as therapeutic targets.^19^ Accordingly, continued research into the biogenesis, functions, and therapeutic potential of circRNAs is anticipated to yield new insights for scientific exploration and medical innovation. In this review, we summarize the expanding findings on circRNAs and provide an up-to-date account of their biogenesis, regulatory mechanisms, and cellular functions in carcinogenesis.

## Biogenesis and functional roles of circRNAs

###  Biogenesis of circRNAs

 In eukaryotic cells, alternative splicing converts pre-RNA into linear mRNA.^16^ On the other hand, circRNAs are formed through aberrant RNA splicing, specifically back-splicing, which is different from canonical splicing. Approximately 80% of circRNAs are derived from exons, but they can also originate from other parts of the genome, like introns, non-coding regions, antisense strands, and untranslated regions (UTRs).^2^ Back-splicing generates numerous different circRNAs from a single gene locus, contributing to the complexity of circRNA formation.^20^ Based on sequence arrangement, circRNAs are classified as exonic circRNAs (ecircRNAs), which contain exon sequences; circular intronic RNAs (ciRNAs), which originate from introns; exonic-intronic circRNAs (EIciRNAs), containing both exonic and intronic sequences; and tRNA intronic circRNAs (tricRNAs), which are formed from spliced tRNA introns.^21-23^ Although the majority of circRNAs reside in the cytoplasm, EIciRNAs mostly remain in the nucleus.^24,25^

 RNA-binding proteins (RBPs) play a crucial role in the regulation of circRNAs synthesis. RBPs like Quaking (QKI), Muscleblind (MBL/MBNL1), and Fused-in Sarcoma (FUS) can bind to specific motifs on the flanking introns of immature linear RNA.^26-28^ These RBPs bring the flanking introns together to facilitate the generation of circRNAs.^29^ Efficient circRNA production requires certain RNA sequence features are needed. For example, exons that can back-splice are often significantly longer up to three times regular exons which is clear in single-exon circRNAs.^30^ Also, the presence of reverse complementary sequences in flanking intronic regions, like Alu elements, enhances intron pairing and exon circularization. These regions can be either longer or shorter than typical introns.^29,31^ Inverted tandem repeats in introns also support circRNA formation, with even short repeats around 35 base pairs being sufficient.^32^ However, these repeats can sometimes make intron base pairing too stable, which makes it less likely for circRNA formation.^33,34^ As circRNAs mature, introns might not always be removed and can stay between the circularized exons, resulting in a subtype of circRNA known as exonic-intronic circRNAs (EIciRNAs).^35^

**Figure 1:**
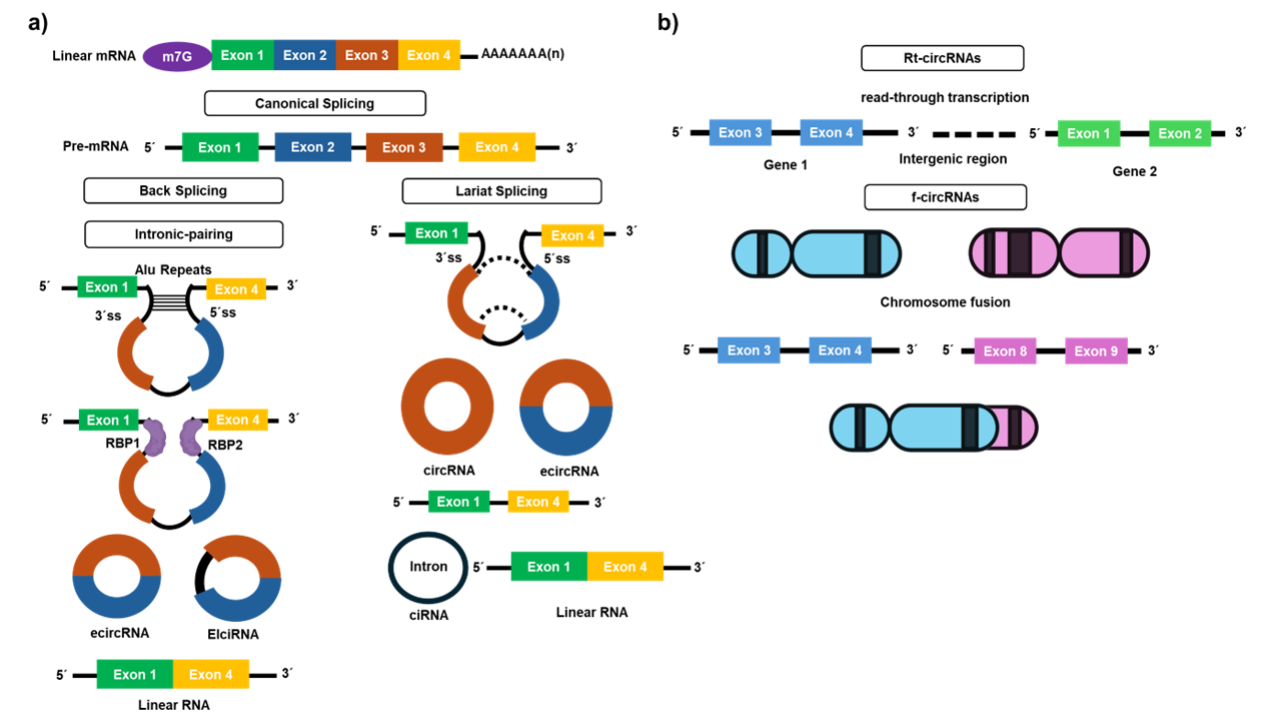
Biogenesis of circular RNAs and new categories of circular RNAs in oncology. (a) Canonical splicing and Primary methods of back-splicing: intron-pairing promoted by inverted complementary sequences and RNA-binding proteins; lariat creation. (b) Production of rt-circRNAs from read-through transcripts. Generation of f-circRNAs by chromosomal fusions

 Despite ongoing research, the exact mechanisms of circRNA biogenesis remain unclear. Three models have been proposed: lariat-driven circularization (exon skipping),^36^ intron pairing-driven circularization,^37^ and re-splicing-driven circularization,^23^ each contributing to our understanding of how these unique RNA molecules are formed. Figure 1a schematically illustrates circRNA biogenesis.One common mechanism is lariat-driven circularization, also known as exon-skipping, where partial folding of pre-mRNA brings the upstream donor site (5’ splice site) and the downstream acceptor site (3’ splice site) into proximity. This allows the donor site to attack the receptor site, resulting in the formation of a lariat structure that is subsequently back-spliced to create a new circRNA. This mechanism is notably stimulated by factors such as tumor necrosis factor (TNF)-α and transforming growth factor (TGF)-β in endothelial cells, producing circRNAs alongside linear mRNA which consists of the remaining exons.^36,38^

 Another key mechanism is intron pairing-driven circularization, which relies on reverse complementary sequences, Alu elements, and the flanking introns. These sequences make possible direct back-splicing. High compatibility between these complementary sequences enhances the circRNA production. This process generates exonic circular RNAs (ecircRNAs) by removing intronic sequences. It also produces exonic-intronic circRNAs (EIciRNAs) that retain some intronic sequences.^37,38^ A third, lesser-known method is resplicing-driven circularization. Here, a mature linear mRNA undergoes back-splicing to produce circRNAs with one or more exons. The concentration of circRNAs within cells is tightly regulated, with their breakdown being crucial for maintaining cellular function. This degradation process involves the partial activation of endonucleases such as Argonaute 2 (Ago-2), Angiogenin, CPSF73, and RNase L, which create access points for exonucleases to degrade circRNAs completely. Each of these pathways underscores the complex and dynamic nature of circRNA biogenesis and its regulation in cellular biology.^23,38,39^

 Cancer may facilitate the development of novel categories of circRNAs, including read-through circRNAs (rt-circRNAs) and fusion circRNAs (f-circRNAs) (Figure 1b). The rt-circRNAs are derived from read-through transcripts. Read-through transcription occurs when transcription extends over an intergenic region beyond the termination signal, resulting in the synthesis of circRNAs from two neighboring genes. Gene pairs that produce rt-circRNAs are shorter than randomly selected neighboring genes pairs. rt-circRNAs share properties with conventional circRNAs, such as elongated introns and an abundance of repetitive motifs. Read-through circularization may be linked to cancer, characterized by widespread abnormal gene expression mediated by transcription read-through. Of the 460 cancer driver genes, 39 were identified to generate 67 rt-circRNAs, with 31 of them exhibiting cancer-specific expression. Nonetheless, their functional importance in cancer requires further confirmation.^40^ Cancer-associated chromosomal translocations may result in the generation of fusion-circular RNAs (f-circRNAs). Aberrant chromosomal rearrangements in malignancies may lead to the juxtaposition of two otherwise separated genes, bringing complementary intronic regions into proximity to facilitate reverse splicing. In 2016, Guarnerio et al initially showed that f-circRNAs originate from PML/RARa fusion mRNAs in acute promyelocytic leukemia and that they contribute to carcinogenesis independently of their linear transcripts and protein equivalents, as well as being associated with resistance to anti-cancer therapy. Subsequent investigations have shown that f-circRNAs arise from specific chromosomal translocations, including BCR/ABL1, EML4/ALK, and SLC34A2/ROS1 fusions, seen in both hematological malignancies and solid tumors.^41^

## Mechanisms of action

**Figure 2:**
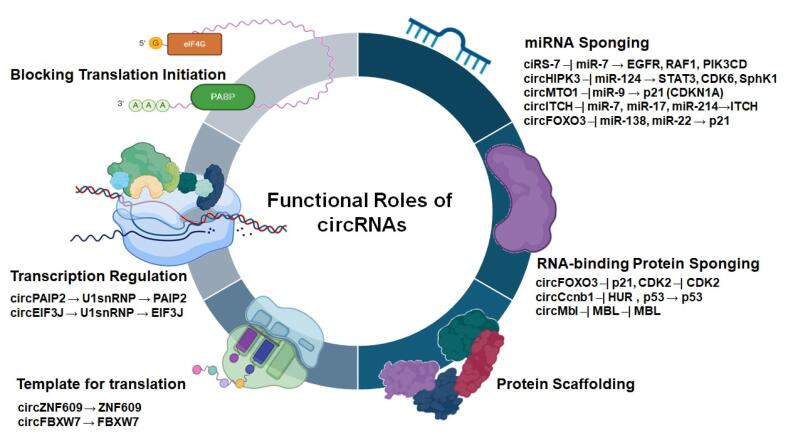
Schematic representation of known functions of circRNAs

 The ability of circRNAs to control gene expression through diverse mechanisms has led to their increasing recognition as important regulators in cancer biology.^42^ Their biogenesis often competes with linear mRNA formation, affecting the production of protein. Functionally, circRNAs regulate gene expression via interactions with miRNAs, RNA-binding proteins, and chromatin, and some even assist as templates for translation (Figure 2). In malignancies, their tissue-specific expression, stability, and subcellular localization contribute to their diverse roles, where they influence metastasis, tumorigenesis, and therapy resistance via tumor-suppressive or oncogenic pathways.

###  miRNA sponging

 CircRNAs are most known for their function as miRNA sponges, which is among their most extensively studied and well-characterized functions. By containing multiple miRNA response elements (MREs), circRNAs can sequester specific miRNAs and suppress them from repressing their target mRNAs, thus controlling gene expression and protein synthesis. This competitive endogenous RNA (ceRNA) activity has an important role in several physiological and pathological processes, as well as malignancies, osteoarthritis, diabetes, and neurological diseases.^43^ For instance, ciRS-7 contains over 70 conserved binding sites for miR-7 and has been shown to inhibit its tumor-suppressive activity in several types of malignancy. Similarly, circHIPK3 which is abnormally expressed in cancer tissues, sponges multiple tumor-suppressive miRNAs such as miR-124, miR-193, and miR-637, thus inducing tumor cell invasion, metastasis and development.^44^ Other circRNAs, as well as circMTO1, circITCH, and circFOXO3, exert tumor-suppressive effects by binding oncogenic miRNAs and regulating key signaling pathways. For example, circMTO1 enhances p21 expression by sponging miR-9 in HCC,^19^ while circITCH and circFOXO3 target miRNAs such as miR-17, miR-214, miR-224, and miR-9 to prevent tumor development and induce apoptosis. In another study, Circular RNA circ-ABCB10 was shown to induce breast tumor proliferation and development via sponging miR-1271.^45^ A comparative summary of notable circRNAs, their miRNA targets, related malignancy types, and functional effects is provided in Table 1 to support and contextualize these findings.

**Table 1:** CircRNAs as miRNA sponges in various cancers

**CircRNA**	**Targeted miRNA**	**Functions**	**Cancer type**	**Ref.**
ciRS-7	miR-7	Myocardial infarction; Neural development; anti-oncogenic; stimulates proliferation/metastasis; osteoblastic differentiation insulin secretion	Various (including breast, liver)	^46^
CircHIPK3	miR-124, miR-193a, miR-558	Stimulates proliferation/migration; prevents cancer progression; β-cell function	Various (including liver, colorectal)	^47^
CircFOXO3	miR-138, miR-9, miR-22	Cell cycle progression and apoptosis; cardiovascular diseases and cancer	Various cancers	^48^
CircZNF91	miR-23b-3p	Mediates signal transduction between hypoxic and normoxic tumor cells to promote pancreatic cancer chemoresistance	Pancreatic cancer	^46^
CircMTO1	miR-9	Prevents cancer progression	HCC	^19^
CircCCDC66	miR-93, miR-185, miR-33b	Stimulates cancer progression	Various (including colorectal)	^49^
circIRAK3	miR-3607	Promotes migration/invasion	Breast cancer	^50^
circRNA_0084043	miR-153-3p	Stimulates cancer progression	malignant melanoma	^51^
CircANKS1B	miR-148a-3p, miR-152-3p	Regulation of TGF-β1 signaling pathway	Breast cancer	^52^
Hsa_circ_0008039	miR-432-5p	Increases E2F3 expression	Breast cancer	^53^
circRNA-000911	miR-499a	Regulation of Notch1 and NF-κB signaling pathway	Breast cancer	^54^
CDR1as	miR-7	Prevention of cell proliferation	Breast, hepatocellular, lung, and gastric cancers	^55,56^
circ-ABCB10	miR-1271	Initiation of cell proliferation	Breast cancer	^45^
circ-ZKSCAN	N/A	Prevention of cell proliferation and metastasis	HCC	^57^
circRNA-100269	miR-630	Prevention of cell proliferation	HCC	^58^
hsa-circ-100338	miR-141-3p	Regulator of metastases	HCC	^59^
hsa_circ_001059	miR-30c, miR-122, miR-139-3p, miR-339, miR-1912	Regulator for tumor radiotherapy resistance	Esophageal squamous cell carcinoma	^60^
circ-ITCH	miR-214	Prevention of cell proliferation by down-regulation of c-myc, ubiquitination, and degradation of Dvl2	Esophageal squamous cell carcinoma, lung cancer, colorectal cancer	^61^
circTCF25	miR-107, miR-103a-3p	Initiation of cell proliferation and metastasis	Bladder cancer	^62^
hsa-circ-0043256	miR-1252	Prevention of cell proliferation	Lung cancer	^63^
Circ-PAX2	miR-186	Initiation of cell proliferation	Lung cancer	^63^
circEA1	miR-372	Regulator for cell differentiation and drug resistance	Lung cancer	^64^
Circ-NFIX	miR-212-3p	Enhances tumor cell progression	Lung cancer	^65^
hsa-circ-001569	miR-145	Initiation of cell proliferation and metastasis	Colorectal cancer	^66^
hsa-circ-0000069	N/A	Initiation of cell proliferation and metastasis	Colorectal cancer	^67^
circPVT1	miR-125	Initiation of cell proliferation and metastasis	Gastric cancer	^68^
circ-LARP4	miR-424-5p	Regulation of tumor progression	Gastric cancer	^69^
circMT01	miR-9	Regulation of tumor progression	HCC	^19^
hsa_circ_000167	miR-181, miR-512, miR-521, miR-556, miR-663 and miR-1204		Esophageal squamous cell carcinoma	^60^
circHIPK3	miR-124	Regulation of tumor proliferation	HCC	^70^
hsa_circ_0067934	miR-98	initiation of cell proliferation	Esophageal squamous cell carcinoma	^71^

###  Protein interaction

 CircRNAs enable directly interact with RBPs, affecting several cellular processes including cancer development and progression by regulating cellular signaling networks. By harboring RBP binding sites, circRNAs function as molecular scaffolds that facilitate or inhibit protein-protein interactions, control RBPs activity, or prevent them from regulating gene expression. Through the formation of ribonucleoprotein complexes, certain circRNAs, such as ecircRNAs, stabilize these interactions and preserve the functional integrity of the related proteins, thus controlling gene regulation at multiple levels.^72^ For example, circ-Foxo3 controls cell cycle arrest by interacting with cyclin-dependent kinase 2 (CDK2) and the protein kinase inhibitor p21. In breast tumor, circ-Foxo3 binds to CDK2 and p21, forming a ternary complex that suppress cell cycle proliferation and tumor development.^73,74^ Additionally, in the context of cellular senescence, circFoxo3 can sequester proteins such as the senescence marker p16 and the transcription factor E2F1, thereby modulating pathways associated with aging and tumor suppression.^48^ In liver malignancy, circ-Ccnb1 interacts with HuR (ELAVL1) to stabilize CCNB1 mRNA, thus inducing oncogenic cell cycle development.^75^ Another example, circMbl, forms a binding interaction with the Muscleblind (MBL) protein, which is a pivotal controller of RNA splicing. During this process, circMbl specifically attracts the MBL protein, which plays a crucial role in the alternative splicing of Mbl pre-mRNA.^76,77^ Furthermore, the decrease of MBL, activation of innate immune dsRNA receptor (PKR) and prevention of Human Antigen R (HuR) protein from binding to Poly(A) Binding Protein Nuclear 1 (PABPN1) mRNA by circPABPN1 can be a result of circRNA activation.^78,79^ In liver cancer, circ-Ccnb1 sponges miR-194-3p, leading to the promotion of matrix metalloproteinase 9 (MMP-9)-mediated oncogenic effects and inducing tumor progression.^80^ These RBP-mediated mechanisms underscore the complexity of circRNA functions in cancer, where protein scaffolding and stabilization roles regulate key tumorigenic pathways.^81^

###  Transcriptional and translational regulation by circRNAs

 In the nucleus, certain circRNAs can to control the gene expression of their host genes. Studies have demonstrated that circRNAs influence the expression of their parental genes through cis-acting mechanisms. In some cases, nuclear circRNAs interact with RNA polymerase II (RNA Pol II) at the promoter region, resulting in the generation of various isoforms of a single gene.^82^ EiciRNAs are the best-known group of circRNAs with transcriptional activity.^83^ As an example, circEIF3J and circPAIP2 regulate the Eukaryotic Translation Initiation Factor 3 Subunit J (EIF3J) and Poly(A) Binding Protein Interacting Protein 2 (PAIP2) gene transcriptions by making a complex with the U1 snRNP. This complex then interacts with RNA Pol II, which regulates the transcription of host genes.^82,84-86^ Circ-ZNF609 and circ-FBXW7 are other examples of transcriptional regulatory circRNAs that are respectively involved in muscular biogenesis and glioma.^87,88^ The binding of circRNAs to RNA Pol II can control selective splicing by regulation of alternative splice site.

 During this process, different splicing sites select pre-mRNAs to produce altered mRNA isoforms. These examples illustrate the diverse roles of circRNAs and highlight their potential as therapeutic targets in cancer and other diseases.^89^ A new study reveals that circRNAs can compete with their host genes in post-transcriptional processes. Additionally, circRNAs possess internal ribosome entry sites, which enable them to translate independently from the host gene. This model is an intelligent way to regulate stability between the expression levels of circRNAs and host mRNAs.^90^ As a result, circRNAs control protein production at the transcriptional or post-transcriptional levels. CircZNF609, c-sirt7, and circMbl are three illustrations of circRNAs with coding probability.^91-93^ In glioblastoma, circFBXW7 is translated into FBXW7-185aa, a peptide that antagonizes c-Myc and prevents tumor cell progression. These findings determine the important role of circRNAs as regulators not only of RNA dynamics but also of protein-coding potential in malignancy.

###  Modulating immunity and metabolism

 Another developing issue is the participation of circRNAs in the immune response. Specific circRNAs can regulate the function of immune cells, thereby impacting the immune system’s ability to react to infections and disorders. This discovery presents new opportunities for investigating circRNAs as potential therapeutic targets for interventions in immune-related diseases.^94^ CircRNAs influence metabolic pathways. They interact with enzymes and other regulatory factors that control metabolism, which can change how cells process food and use energy. This affects processes such as maintaining blood sugar levels, metabolizing fats, and producing energy. Because circRNAs can do this, they may play a role in health problems related to metabolism, such as obesity and diabetes.^95^ As we learn more about circRNAs, we can see that these molecules play a key role in how cells control themselves. They can interact with multiple targets within the cell, and they are characterized by high stability and specificity. This property makes circRNAs promising candidates for diagnosing and treating diseases. As we continue to study them and our technology improves, we will learn even more about the function of circRNAs. This will pave the way for novel discoveries and significant advancements in the field of biomedical science.^96^

## Techniques for measuring circRNAs

**Figure 3:**
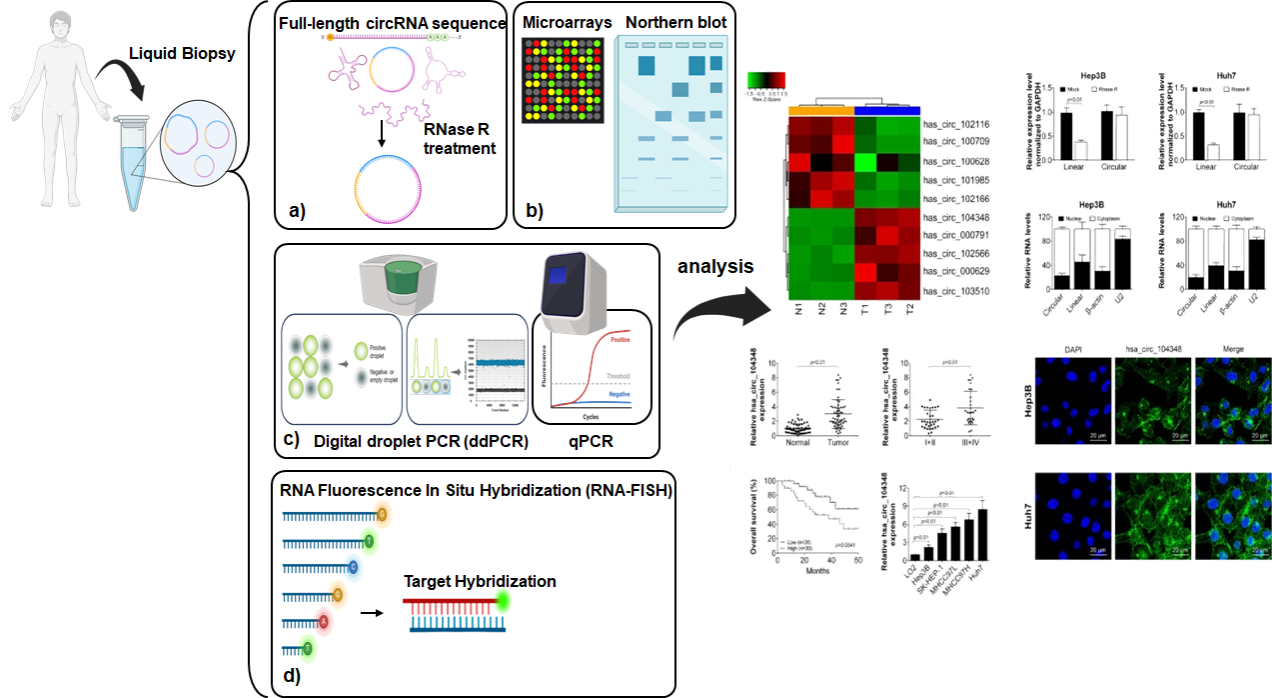
Schematic representation of diverse circRNA profiling methods

 Measuring and evaluating circRNAs requires special methods because of their unique closed-loop structure, which makes them different from linear RNAs. CircRNA sequencing is a common technique that begins with RNase R treatment to degrade linear RNAs, facilitating the process of circRNA sequencing. This step is crucial as it leaves the circular RNAs intact, allowing for their identification based on unique back-splice junctions through high-depth sequencing, although RNAase treatment is not always mandatory (Figure 3a).^22,97-99^ CircRNA microarrays offer another high-throughput approach by using probes specifically designed to hybridize with the junction sequences of circRNAs on a solid surface, providing a robust platform for evaluating circRNA expression without requiring RNase treatment, although this can improve accuracy.^97,100,101^ Northern blotting stands out because it can provide detailed information about the size, isoforms, processing, sequence, and abundance of circRNAs (Figure 3b). It distinguishes between circRNAs and linear RNAs by using different gel electrophoresis methods based on the size of the RNAs.^97,102^

 Real-time quantitative polymerase chain reaction (RT-qPCR) analysis utilizes distinct primers that cover the unique back-splice junctions of circRNAs, enabling accurate quantification while preventing the amplification of linear RNAs. This method can optionally use RNase treatment to increase the concentration of circRNAs, thereby improving measurement accuracy.^103^ Digital droplet PCR (ddPCR) provides exceptional sensitivity for circRNA quantifying, utilizing nanodroplets for PCR amplification, and determining RNA concentration by comparing the ratio of positive to negative droplets. This method is highly accurate even for low-abundance circRNAs (Figure 3c).^104,105^

 For spatial analysis, RNA Fluorescence in situ Hybridization (RNA-FISH) utilizes probes to detect circRNA junctions. To assess their concentration and distribution inside cells, showing their cellular localization and dynamics (Figure 3d).^106^ Additionally, to explore how circRNAs interact at the molecular level, methods such as circRNA affinity pulldown, which utilizes biotinylated antisense oligomers (ASOs) to capture circRNAs with streptavidin-coated beads, facilitating interaction mapping within the molecular network. Similarly, immunoprecipitation of circRNA-RBP complexes isolates circRNA-protein complexes using antibodies that target RNA-binding proteins associated with circRNAs. This step allows for later analysis of the RNA component using ddPCR and RT-qPCR. These different methods enhance our understanding of the expression, structure, and functional roles of circRNAs in cellular biology, as well as their potential therapeutic applications.^22,97,107^

###  Diagnostic and prognostic potential of circRNAs in cancer

**Figure 4:**
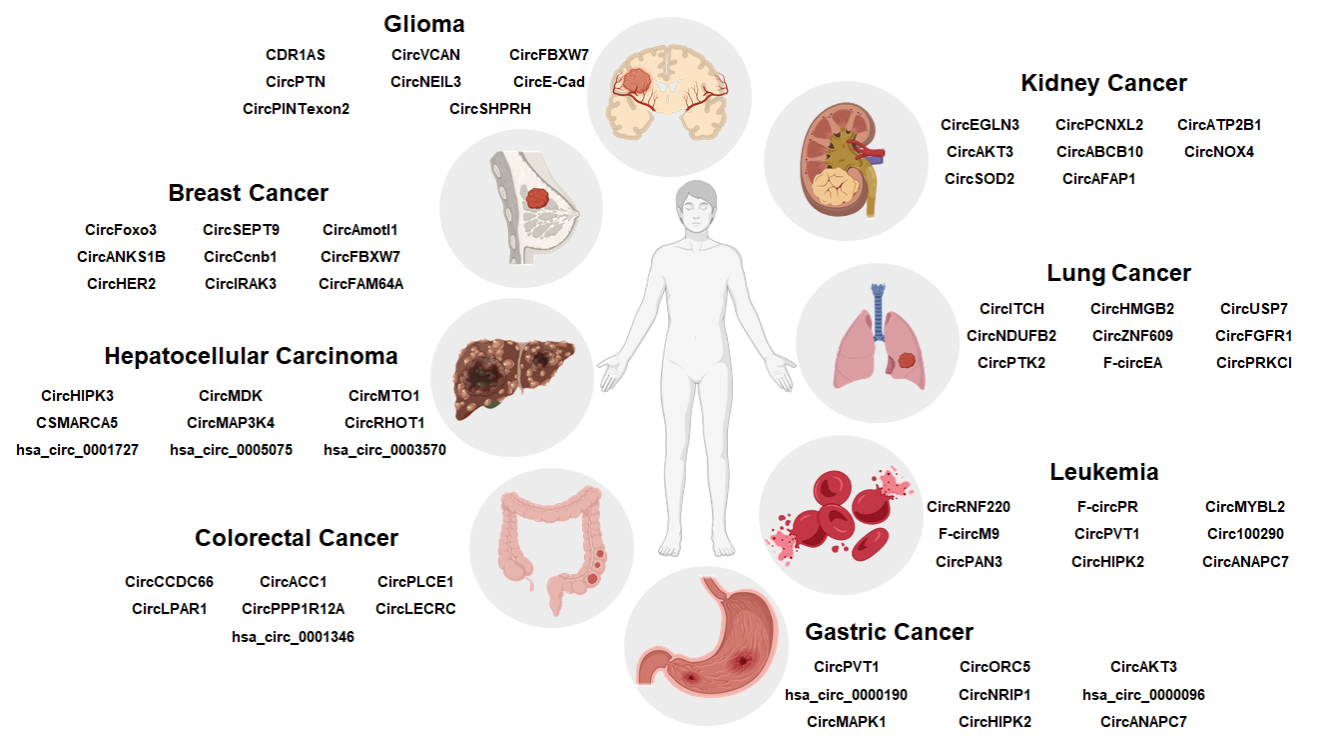
CircRNAs as biomarkers and therapeutic targets in cancer.This diagram depicts the role of circular RNAs (circRNAs) as biomarkers in cancer diagnosis. The human silhouette in the center represents the patient. Different circRNAs are connected to specific cancer types. These circRNAs enable diagnostic outcomes, such as early detection of tumors, monitoring of tumor progression, and evaluation of treatment responses. The illustration emphasizes the potential of circRNAs as non-invasive biomarkers in liquid biopsy applications for cancer diagnosis

 circRNAs have gained attention as important diagnostic and prognostic biomarkers in various malignancies due to their remarkable stability, abundance in body fluids such as plasma and serum, tissue-specific expression pattern, and enabling non-invasive detection (Figure 4).^108^ Unlike linear RNAs, which typically degrade within 20 hours, the half-life of circRNAs in body fluids generally exceeds 48 hours, which is significantly longer than the half-life of linear RNAs that related to their closed-loop structure and protection within extracellular vesicles. Recent research mention that the half-lives of circRNA vary between 8 and 50 hours, depending on the specific circRNA species and contexts.^109^ Several researches have shown that expression levels of certain circRNAs are related with clinicopathological characteristics including migration potential, tumor stage, and tumor size, connecting their dysregulation to tumor invasion and development.^110^ Recent Meta-analyses have demonstrated that circRNAs show good diagnostic performance, with pooled sensitivity and specificity of around 79% and an area under the curve (AUC) of approximately 0.86 in hematological malignancies, demonstrating their potential for early malignancy detection and support clinical management.^111^ Moreover, combining multiple circRNA into panels considerably improves diagnostic accuracy compared to single circRNAs. For instance, in gastric cancer, a combination of circRNAs improved the AUC from 0.82 to 0.91, representing better sensitivity and specificity.^112^

 Similarly, a classifier that employs five circRNAs (circPDLIM5, circSCAF8, circPLXDC2, circSCAMP1, and circCCNT2) extracted from urine extracellular vesicles has shown acceptable performance to recognize high-grade prostate cancer of grade 2 or above.^113^ In pancreatic ductal adenocarcinoma (PDAC), a panel of five plasma-based liquid biopsy circRNAs (hsa_circ_0060733, hsa_circ_00061117, hsa_circ_0064288, hsa_circ_0007895, and hsa_circ_0007367) was able to distinguish between early stage (stage I/II) and late stage (stage III/IV). Diagnostic accuracy of this circRNA panel for detecting PDAC patients was considerably increased and improved when combined with cancer antigen 19-9 (CA19-9), the conventional biomarker for PDAC.^114^ On the prognostic side, increased levels of oncogenic circRNAs are associated with poorer overall survival (hazard ratios ranging from 1.3 to 2.3), whereas tumor-suppressive circRNAs are often correlated with better survival rates. These findings underscore circRNAs value in predicting patient prognosis and therapeutic response. Despite these hopeful findings, several challenges and limitation remain, including the lack of standardized detection techniques, large-scale validation studies, and mechanistic insights to fully integrate circRNAs into clinical practice as reliable biomarkers for cancer diagnosis and prognosis are remain.^81^ hsa-circ-0001649 and hsa-circ-002059 have shown strong potential as biomarkers in human HCC.^115,116^ Similarly, hsa-circ-001988 has been proposed as a diagnostic marker for gastric cancer.^117^ While, circPRMT5 was introduced as a biomarker for urothelial carcinoma, where it appears have a role in tumor development and lymph node metastasis.^118^ Table 2 summarizes circular RNAs that highlights potential biomarkers in several cancer types. As discussed in previous parts of this review, circular RNAs have four recognized functions, and all of them are important in malignancy development.^119^ Furthermore, circRNAs employ as predictive markers for malignancy treatment efficacy and resistance. Several circRNAs have been recognized for their role in controlling chemosensitivity, underscoring their value in guiding treatment strategies.

**Table 2:** CircRNAs with prognostic values in cancers.

**circRNA**	**Cancer**	**Model Used**	**Mode of Action**	**Translational Stage**	**Related pathological features**	**Ref.**
circPRMT5	Urothelial carcinoma	In vitro (UCB cell lines), In vivo (BALB/c nude mice xenograft metastasis model)	Sponging miR-30c/upregulates SNAIL1/induces/EMTl promotes metastasis	Preclinical (patient tissue, mouse, exosome-based biomarker)	Tumor progression and lymph node metastasis	^118^
hsa_circ_0001874	OSCC	Human salivary samples (clinical patient cohort, 90 OSCC vs. 82 controls)	miRNA sponge (targets: miR-661, miR-662, miR-593-5p, miR-107, miR-103a-3p); linked to TNM stage and tumor grade	Biomarker validation	Tumor progression, TNM stage, tumor grade	^120^
hsa_circ_0001971	OSCC	Human salivary samples (clinical patient cohort, 90 OSCC vs. 82 controls)	miRNA sponge (targets: miR-152-5p, miR-103a-3p, miR-107, miR-505-3p, miR-9-5p); associated with TNM stage	Biomarker validation	Tumor progression, TNM stage, tumor grade	^120^
hsa-circ-0001785	Breast cancer	Human plasma samples (n = 57 patients)	Presumed miRNA sponge (not directly confirmed); associated with tumor burden	Biomarker validation	TNM stage, histological grade, distant metastasis, surgery response	^121^
Circ-ZEB1.33	HCC	Human HCC tissues, adjacent non-tumorous tissues, HCC cell lines	miRNA sponge for miR-200a-3p/upregulation of CDK6/ enhanced cell proliferation	Preclinical functional studies	Tumor proliferation, upregulated in HCC tissues vs. normal	^122^
hsa_circ_0005075	HCC	94 paired HCC and adjacent normal tissues; clinicopathological correlation	Downregulated in HCC; correlated with tumor size and AFP level (suggestive biomarker role)	Pre-clinical	Tumor size, AFP level, Edmondson stage, poor differentiation in HCC	^123^
hsa-circ-0001649	HCC	60 paired HCC and adjacent non-tumor liver tissues; cell lines	Downregulated; ROC analysis suggests potential diagnostic biomarker	Clinical correlation + biomarker discovery	Tumor size, TNM stage, AFP levels; diagnostic potential (AUC: 0.834)	^115^
hsa_circRNA_100855	LSCC	Human LSCC tissues (n = 52 paired samples), qRT-PCR, microarray	Not mechanistically tested	Clinical association study	Overexpressed in LSCC; correlated with T3–T4 grade, lymph node metastasis, supraglottic site, and advanced stage	^124^
hsa_circRNA_104912	LSCC	Human LSCC tissues (n = 52 paired samples), qRT-PCR, microarray	Not mechanistically tested	Clinical association study	Downregulated in LSCC; correlated with T3–T4 grade, lymph node metastasis, poor differentiation, and advanced stage	^124^
hsa_circ_0003221 circPTK2	Bladder cancer	40 paired patient tissues and blood samples - Human cell lines (T24, 5637) - Nude mice xenograft model	Promotes proliferation and migration; potentially via miRNA sponge (mechanism not fully defined)	Preclinical (in vitro & in vivo)	Overexpressed in poorly differentiated tumors, advanced T stage (II–IV), and N2–N3 lymph node metastasis	^125^
hsa-circ-001988	Colorectal cancer	31 paired colorectal tumor and adjacent normal tissues - Clinical correlation with patient data	Downregulated in CRC; associated with poor differentiation and perineural invasion; possible biomarker	Preclinical sample-based study	Decreased expression in tumors; linked to tumor differentiation, perineural invasion; ROC AUC = 0.788 for diagnosis	^126^
hsa_circ_002059	Gastric cancer	Human gastric cancer tissues and plasma	Downregulate expression; potential biomarker; associated with TNM stage and metastasis	Preclinical diagnostic biomarker study	Associated with TNM stage, distal metastasis	^127^

Abbreviations: OSCC, Oral squamous cell carcinoma; HCC, hepatocellular carcinoma; LSCC, Laryngeal squamous cell carcinoma.

 For instance, a specific circRNA expression signature has been used to predict the response to immune checkpoint blockade therapy. The ICBcircSig score was validated based on the weighted expression of circTMTC3 and circFAM117B from melanoma in patients receiving anti-PD-1 or combined anti-CTLA4 and anti-PD-1 therapy. This model, representing that the ICBcircSig score has valuable role in predicting immunotherapy efficacy in melanoma patients.^128^

 A growing number of clinical trials are evaluating the potential of circRNAs as diagnostic and prognostic biomarkers in several malignancies (Table 3). For instance, trial NCT05771337 is recruiting breast tumor patients to validate the clinical efficacy of two plasma-based circRNAs (hsa_circ_0001785 and hsa_circ_100219) for early detection, diagnosis and disease monitoring.^129^ Additionally, NCT06530082 is evaluating a new dendritic cell vaccine based on a circRNA-derived peptide (circFAM53B-219aa) in individuals with advanced solid tumors (https://www.careacross.com/clinical-trials/trial/NCT06530082). In pancreatic cancer, the CIRCUS trial is exploring circRNA panels for early diagnosis and comparing their efficacy with standard markers, such as CA19-9.^114^ Furthermore, pilot studies are exploring exosomal circular RNAs in cerebrospinal fluid as potential markers for tracking glioma recurrence.^130^ Also, preclinical studies are evaluating circPVT1 as a predictor of drug resistance in estrogen receptor alpha-positive (ERα + ) breast cancer.^131^ Collectively, these researches highlight the growing clinical interest in circRNAs as minimally invasive, stable, sensitive, and specific biomarkers for cancer diagnosis, prognosis, and therapeutic monitoring.

**Table 3:** Clinical trials investigating circRNAs as biomarkers in cancers

**Trial**	**Cancer type(s)**	**circRNA(s) studied**	**Approach/Goal**	**Status**	**Ref.**
NCT05771337	Breast cancer	hsa_circ_0001785, hsa_circ_100219	Diagnostic/prognostic validation in plasma/serum	Recruiting	^132^
NCT06530082	Advanced solid tumors	circFAM53B-219aa (peptide vaccine)	Immunotherapy, safety, and efficacy assessment	Phase I/II, ongoing	^129^
CIRCUS Trial	PDAC	circPDE8A, circRHOBTB3, panel	Early detection, comparison with CA19-9	Preclinical/Clinical	^114^
Glioma (pilot studies)	Glioma	circSMARCA5, circHIPK3 (exosomal)	Recurrence monitoring in CSF	Preclinical	^130^
Drug resistance (preclinical)	Breast cancer (ERα + )	circPVT1	Predicting tamoxifen resistance	Preclinical	^131^

Abbreviation: PDAC, Pancreatic ductal adenocarcinoma.

## Therapeutic applications of circRNAs in cancer treatment

 Therapeutic approaches that focus on circRNAs present a hopeful and innovative future in the field of cancer treatment. circRNAs possess distinct characteristics, such as their exceptional stability and specific interactions with miRNAs and proteins, which render them highly suitable for therapeutic intervention.^133^ To further clarify the translational relevance of circRNAs in cancer, Table 4 provides a comparative overview of well-characterized circRNAs, detailing their associated cancer types, experimental models, modes of action, and current translational status. Here, we examine various strategies that have been developed to utilize circRNAs for cancer treatment.

**Table 4:** Comparative summary of promising circRNAs across cancer types: experimental models, molecular mechanisms, and translational relevance

**circRNA**	**Cancer type(s)**	**Model used**	**Mode of action**	**Translational stage**	**Function**	**Ref.**
CircHIPK3	Colorectal	In vitro	Sponges miR-1207-5p; upregulates FMNL2; promotes proliferation and metastasis	Preclinical (in vitro, tissue analysis)	Oncogene	^134^
circHIPK3	Bladder	In vitro (T24T, UMUC3); In vivo (xenograft, metastasis model in nude mice)	Sponges miR-558, downregulating heparanase (HPSE), inhibits MMP-9, VEGF, suppresses angiogenesis and metastasis	Preclinical (in vitro + xenograft mouse model)	Tumor suppressor	^135^
circMTO1	HCC	In vitro (HepG2, SMMC-7721, QGY-7701, SK-Hep1); In vivo (SMMC-LTNM xenograft in nude mice); Human tissue	Sponges miR-9; increases p21 expression; tumor suppressor	Preclinical (in vitro, in vivo, and clinical tissue)	Tumor suppressor	^19^
circITCH	ESCC	In vitro (Eca-109, TE-1); In vivo (xenograft nude mouse); Clinical tissues	circITCH acts as a sponge for miR-7, miR-17, and miR-214. suppression of the Wnt/β-catenin signaling pathway	Preclinical (cell lines and mouse xenografts)	Tumor suppressor	^136^
CDR1as (ciRS-7)	HCC	In vitro (HepG2, MHCC-97H); In vivo (xenograft mouse);	circRNA Cdr1as sponges miR-1270, promotes proliferation, migration	Preclinical (in vitro + in vivo)	Oncogene	^137^
CDR1as (ciRS-7)	Breast cancer triple-negative breast cancer	In vitro (MDA-MB-231, BT-549); In vivo (nude mouse tail vein metastasis model)	Sponges miR-1299, upregulates MMP2 and MMP17,promotes migration, invasion, and metastasis	Preclinical (in vitro, in vivo, and patient tissues)	Oncogene	^138^
circSMARCA5	Prostate cancer	In vitro (DU145, PC3, LNCaP); In vivo (xenograft and metastasis mouse models); Clinical tissues	Sponges miR-181b-5p and miR-17-3p upregulate TIMP3, inhibit EMT, proliferation, invasion, metastasis	Preclinical (in vitro + in vivo + tissue validation)	tumor suppressor	^139^
circFBXW7	Glioma	In vitro (U251, U373); In vivo (xenograft nude mice); Clinical samples	Encodes FBXW7-185aa protein; promotes c-Myc degradation via USP28 competition	Preclinical (in vitro, in vivo, and clinical tissue)	Tumor suppressor	^140^
circFOXO3	Non-small cell lung cancer	In vitro	Sponges miR-155, Upregulates FOXO3, a tumor suppressor, inhibits proliferation and invasion	Preclinical (in vitro + human tissue)	Tumor suppressor	^141^
circFOXO3	Prostate cancer	In vitro (LNCaP, PC-3, DU145, 22Rv1)	Sponges miR-29a-3p, upregulates SLC25A15, promotes proliferation, suppresses apoptosis	Preclinical (in vitro + clinical tissue)	Oncogene	^142^
circRNA_100290	Colorectal cancer	In vitro (HCT116, SW620)	Sponges miR-516b, upregulating FZD4, activates Wnt/β-catenin signaling, promotes proliferation, invasion, migration	Preclinical (in vitro + clinical tissue analysis)	oncogene	^143^
circPVT1	Gastric cancer	In vitro (AGS, SGC-7901)	circPVT1 functions as a competing endogenous RNA (ceRNA) by sponging miR-423-5p	Preclinical (in vitro)	Oncogene	^144^
circPVT1	Osteosarcoma	In vitro (U2OS, MG63); Clinical tissue	Sponges miR-205-5p indirectly upregulates c-FLIP, promotes EMT, invasion, and metastasis	Preclinical (in vitro + human tissue)	Oncogene	^145^
circRNA_0025202	Breast	In vitro (MCF-7, MCF7/TR, T47D); In vivo (xenograft nude mice); Clinical samples	Sponges miR-182-5p; upregulates FOXO3a; suppresses tumor growth	Preclinical (in vitro + in vivo + clinical tissues)	Tumor suppressor in HR + breast cancer	^146^
circGFRA1	Triple-negative breast cancer	In vitro (MDA-MB-231, BT549, MDA-MB-468); In vivo (xenograft mouse model); Clinical tissues	Sponges miR-34a, upregulates GFRA1, promotes proliferation, suppresses apoptosis, linked to poor prognosis	Preclinical (in vitro + in vivo + patient samples)	Oncogene	^147^
circUHRF1 (hsa_circ_0048677)	HCC	In vitro (HCC cell lines + NK-92 cells), In vivo (xenograft in NOD/SCID mice)	Sponges miR-449c-5p → upregulates TIM-3 on NK cells → → induces NK cell exhaustion	Preclinical validation and retrospective clinical association with anti-PD1 resistance	Oncogene	^148^
circPRKCI	Lung adenocarcinoma	In vitro (LUAD cell lines)/In vivo (xenograft)	Sponges miR-545 and miR-589, leading to upregulation of E2F7, promoting cell proliferation and tumorigenesis	Preclinical	Oncogene	^149^
circZNF609	Breast cancer	In vitro (MCF7, MDA-MB-231); In vivo (nude mouse xenograft)	miRNA sponge (miR-145-5p), upregulates p70S6K1, promotes proliferation, migration, invasion	Preclinical	Oncogene	^150^

Abbreviations: ESCC, Esophageal squamous cell carcinoma; HCC, hepatocellular carcinoma; FMNL2, formin-like 2; CDR1as, Cerebellar degeneration-related protein 1 antisense RNA; ceRNA, endogenous RNA; OSCC, Oral squamous cell carcinoma; LUAD, Lung adenocarcinoma; EMT, Epithelial-mesenchymal transition.

## circRNAs targeting strategy

###  Antisense oligonucleotides (ASOs)

 One of the prominent approaches is the use of antisense oligonucleotides (ASOs) specifically degrade oncogenic circRNAs. ASOs are synthetic, short nucleotide sequences that regulate the function of target genes. The FDA has approved two ASO drugs for Duchenne muscular dystrophy (DMD) and spinal muscular atrophy (SMA) treatment.^151^ circHIPK3, which promotes colorectal and esophageal cancer (EC) by sponging tumor-suppressive microRNAs, can be targeted by ASOs to disrupt its oncogenic activity and restore the normal function of these microRNAs. This targeted degradation can significantly inhibit tumor growth and progression.^18,152^ Chemical modifications such as phosphorothioate backbones and 2’-O-methylation increase nuclease resistance while simultaneously decrease immunogenicity. Despite these advancements, challenges related to delivery efficiency and tissue specificity remain important problem to clinical translation. Advances in circRNA annotation, modify chemical of ASOs, and improvement of targeted delivery systems may enable for the development of circRNA-directed precision therapeutics that control circRNA activity in a selective and effective way.^153^ In a pivotal study, Legnini et al confirmed that circZNF609 can be selectively silenced using BSJ-specific ASOs, which led to decrease myoblast proliferation without altering the linear transcript.^91^

###  CRISPR/Cas9 gene editing

 Advanced gene editing technologies such as CRISPR/Cas9 offer the potential to correct dysfunctional circRNAs implicated in cancer. This approach can be employed to delete or modify oncogenic circRNAs, such as circPRKCI in lung adenocarcinoma, thereby restoring normal cellular functions and inhibiting cancer progression.^149^ CRISPR/Cas9-mediated knockout of specific circRNAs can provide insights into their roles in malignancy and pave the way for targeted therapies.^154^ The application of CRISPR/Cas9- to target circRNAs shows hopeful new avenue for cancer treatment, particularly in tumors where specific circRNAs are involved in malignancy development, treatment resistance, or migration potential. Since circRNA expression profiles can vary between different tumor types and even among individual patients, CRISPR/Cas9 technology aligns well with the principles of personalized medicine. By using CRISPR technology to target specific circRNAs correlated with poor prognosis or therapeutic resistance, it may be possible to develop highly specific, patient-centered interventions.^155^ In related work, CRISPR/Cas9 system has been planned to target the telomerase reverse transcriptase (TERT) promoter or its coding regions. These interventions have effectively prevented TERT transcription and decrease telomerase activity which leading to suppressed cell progression and development and increased apoptosis in malignancy cells.^156^ As of May 2025, no clinical trials have been published, particularly using CRISPR technology to target circRNAs. However, several preclinical investigations have made considerable progress. Notably, Zhang et al developed an improved CRISPR/Cas13d platform that efficiently degrades circRNAs at the RNA level with higher specificity and efficiency than shRNA methods.^157^

## Delivery strategies

###  Nanoparticle-based delivery systems

 Another promising strategy involves the engineering of circRNA-based nanoparticles for targeted drug delivery. These nanoparticles encapsulate circRNAs, shielding them from nuclease-mediated degradation and enabling their effective delivery into tumor cells. Among the several platforms, lipid nanoparticles (LNPs) has been particularly successful in targeting tumors, types, especially in HCC.^158,159^ In preclinical studies, LNPs loaded with circRNAs promoted apoptosis, and prevent tumor cell proliferation and progression.^160,161^ You et al designed magnetically responsive nanoplatforms based PEG-PCL-PEI-C14-coated superparamagnetic iron oxide nanoparticles (SPIONs) for delivering siRNA to target circ_0058051, which led to considerable decrease in circRNA expression and noteworthy tumor inhibition in a HCC model, with no observable off-target toxicity.^162^ Similarly, Shu et al. used chitosan–epigallocatechin gallate (CS–EGCG) nanoparticles to deliver a circSPIRE1 overexpression plasmid via systemic administration. Their finding showed a significant decrease in lung metastasis in renal cell carcinoma model, by promoting epithelial integrity and repressing angiogenesis.^163^

###  Exosome-based delivery systems

 Exosome-based delivery systems have garnered important attention as a next generation platform to developing circRNA therapeutics. These nano-sized extracellular vesicles, naturally secreted by cells, can be designed to carry circRNAs directly to tumor cells. These exosomes offer several advantages, as well as their innate ability to pass biological barriers and target specific tissues, such as malignant cells. For instance, circ-0025202 was successfully delivered to breast cancer cells via engineered exosomes. This approach led to considerable decrease in tumor progression and metastasis, indicating the therapeutic potential of exosome-mediated circRNA delivery. This therapeutic strategy utilizes the natural stability and efficient cellular uptake mechanisms of exosomes to promote circRNA delivery while reducing toxicity.^164^

## Synthetic circRNAs and tumor suppressor restoration

###  Synthetic circRNAs

 Synthetic circRNAs can be designed to act as sponges for oncogenic miRNAs, preventing these miRNAs from promoting tumor growth and resistance to apoptosis. For example, synthetic circRNAs can be designed to sequester miR-21-5p, an oncogenic microRNA in cancer cells, thereby preventing it from promoting tumor growth and resistance to apoptosis. These synthetic circRNAs can reduce miRNA availability, thus increasing the tumor-suppressive gene expression like RECK and PDCD4.^165,166^

###  Upregulating tumor suppressor circRNAs

 CircRNAs like circITCH have confirmed tumor-suppressive function by controlling important oncogenic pathways. In bladder cancer, circ-ITCH prevents tumor growth and development by sponging miR-17/miR-224 and leading to increased expression including p21 and PTEN.^167^ In colorectal tumor, circITCH suppresses cell progression and growth via sponging miR-7, which results in elevated expression ofITCH, a known negative controller of theWnt/β-cateninsignalingpathway.^168,169^ Likewise, in gastric cancer, both in vitro and in vivo research have confirmed that cir-ITCH have a tumor suppressor function to suppress tumor carcinogenesis by binding to miR-17 and subsequently downregulating the Wnt/β-catenin pathway.This suppressive impact on tumor progression and growth was decreased when miR-17 was reintroduced.^170^ Despite these hopeful preclinical results, high expression of tumor suppressor circRNAs for treatment goals have several technical and translational challenges. Improvement invector engineering, nanoparticle-baseddelivery system, and chemical modifications are being developed to overcome these barriers and enable the transition of circRNA-based tumor suppressors into clinical applications.^171^ Table 5 showstumor suppressor circRNAs and preclinical upregulation strategies.

**Table 5:** Tumor suppressor circRNAs and preclinical upregulation strategies

**circRNA**	**Cancer type**	**Mechanism**	**Therapeutic strategy**	**Effect**	**Clinical/preclinical**	**Ref.**
circSMARCA5	Glioblastoma	Sponges oncogenic miRNAs; inhibits angiogenesis	Plasmid overexpression / synthetic circRNA	↓ Proliferation, ↓ Migration	preclinical	^173^
circFOXO3	NIH3T3 cell, B16 cells (mouse melanoma cell line)	Binds CDK2/p21; blocks cell cycle progression	Viral vector overexpression	↑ Apoptosis, ↓ Tumor growth	preclinical	^174^
circITCH	Colorectal, bladder	Sponges miRNAs regulating Wnt pathway	plasmid-based overexpression, Lentiviral delivery	↓ Wnt signaling, ↓ Cell proliferation	preclinical	^168,170^

## Immune modulation

 Circular RNAs are emerging as both modulators and potential targets in cancer immunotherapy. Moreover, synthetic circRNAs can be designed to modulate the immune response against cancer. These synthetic circRNAs can act as decoys for immune checkpoint proteins, such as PD-L1, thereby enhancing the body’s immune response against tumors.^174^ Collectively, these therapeutic strategies underscore the versatile potential of circRNAs in cancer treatment, offering novel avenues to target and overcome the molecular complexities of cancer. Table 6 presents the circRNAs involved in immune modulation and their therapeutic potential.

**Table 6:** circRNAs involved in immune modulation and therapeutic potential

**circRNA**	**Cancer type**	**Immune function**	**Therapeutic strategy**	**In vitro/in vivo**	**Ref.**
circUHRF1	HCC	Induces NK cell exhaustion via TIM-3 upregulation	lentiviral shRNA system + anti-PD1	in vitro and in vivo	^149^
circ-CPA4	NSCLC (A549 and H1299 cell lines; BALB/c nude mice xenografts)	Promotes PD-L1 expression; immune evasion	siRNA and shRNA-mediated knockdown	in vitro and in vivo	^176^
circEIF3K	colorectal cancer (HCT116, SW620, FHC)	Silencing circEIF3K, up-regulate miR-214, reducing PD-L1 expression	siRNA and lentiviral shRNA	in vitro and in vivo	^177^

###  circRNAs as immune modulators in the tumor microenvironment

 Recent research indicates that certain circRNAs control immune checkpoints, cytokine production, T-cell activity, and immune cell infiltration, thus can contribute to tumor immune evasion and escape. One well-studied example is circUHRF1, which is notably highly expressed in HCC, is secreted via exosomes. This circRNA promotes NK cell exhaustion by increase in T-cell Immunoglobulin and Mucin-domain containing-3 (TIM-3) expression. Functional studies have confirmed that knockdown of circUHRF1 restores NK cell cytotoxicity and enhances the efficacy of anti-programmed cell death protein-1 (PD-1) therapy, underscoring its potential as a therapeutic target.^148^ Similarly, in non-small cell lung cancer, circ-CPA4 facilitates immune escape by spongingmiR-377, resulting in overexpression of PD-L1 and immune evasion and escape. Knockdown of circ-CPA4, sensitize tumors to immune checkpoint blockade, further highlighting the clinical importance of circRNAs in regulating immune responses.^175^

###  circRNAs as biomarkers for immunotherapy response 

 CircRNAs exhibit exceptional stabilityin blood and exosomes, making them attractive candidates for non-invasive biomarkers to predict immunotherapy response. A compelling example is exosomalcircEIF3K, which is derived from cancer-associated fibroblast induces colorectal cancer growth by the miR-214/PD-L1 signaling pathway.^176^

## Clinical challenges in circRNA-based therapeutic

 With pay attention to the acceptable promise of circRNA-based therapeutics, the application of circRNAs stay in preclinical phase, introducing these challenges is important for proceeding their development from preclinical studies to clinical applications. This section highlights the important limits of these techniques and explores potential strategies for overcoming them. One important dis-advantage is the risk of off-target gene silencing, where RNA interference (RNAi) techniques including the use of small interfering RNAs (siRNAs), can knockdown unintended genes due to partial complementarity leading to unexpected and mostly damaging effects. Although recent techniques, like CRISPR/Cas13 technology, show higher specificity in targeting circRNAs, but these method and technology still require validation *in vivo* application, before they can be safely used in clinic.^177^ Another considerable challenge is related the non-specific delivery of therapeutic agents to tissues or cell types since some circRNAs are expressed in multiple tissue types, this can lead to off-target effects in non-diseased tissues. For this reason, researchers are designing nanoparticle delivery platform that induce the selectivity and accuracy of delivering therapeutic agents to specific tissues or cell.^178^ Furthermore, numerous technical and safety problems prevent the clinical translation of circRNA-based therapies. For instance, gold nanoparticles (AuNPs), widely used to deliver vehicles for circRNA-targeting agents in vivo models whereas increase toxicity and safety risks.^179^ Ongoing research is focused on optimizing AuNP properties for improve safety and biocompatibility or selecting safer alternatives, including lipid nanoparticle (LNP)-based systems, which are already approved for use in mRNA-based vaccines and RNA therapies.^178^ However, the using LNPs also has some disadvantages, including inefficient endosomal escape and limited ability to target solid tumors is the main reason to prevent their application in malignancy treatment therapy.^180^

 In clinical scale, another important disadvantage is the complexity and cost of producing high-purity circRNAs. High expression vectors frequently generate linear or mis-spliced byproducts, decreasing therapeutic purity. Recent studies in template-based in vitro circularization and purification are hopeful but they are not yet suitable for industrial production.^181^ Moreover, synthetic circRNAs may induce immune responses because they do not have specific post-transcriptional modifications present in endogenous circRNAs, including N^6^-methyladenosine (m^6^ A), which help them to evade immune responses. There are some techniques to decrease synthetic circRNAs immunogenicity including chemical modifications and coating synthetic circRNAs with RBPs to escape immune responses.^182^ From a diagnostic feature, the remarkable stability of circRNAs in body fluids like plasma, serum, and saliva supports their potential application in liquid biopsy platforms. However, the clinical applications of circRNAs stay limited due to the absence of standardized detection protocols and unpredictable functional validation in different patient cohorts. Moreover, ongoing discussion about the coding potential of some circRNAs, emphasizes the need for more complete functional characterization and mechanistic studies.^183^ Recent studies have also underscored translational setbacks. For instance, overexpression of circRNAs leads to induction of immune responses or inability to reproduce in vitro effects in vivo. to address this challenge, researchers are engineering synthetic circRNAs that more closely mimic endogenous molecules, with the goal of maintaining function and reducing immunogenicity. Additionally, combination therapeutic methods, including pairing circRNA delivery with immune checkpoint inhibitors, are also being investigated to increase efficiency.^184^ Overcoming these challenges is essential to translating the preclinical findings into effective clinical therapies.

## Conclusion

 circRNAs are gaining recognitionas important regulators in cancer biology, with diverseroles as diagnostic biomarkers, therapeutic targets, and even direct treatment approaches. Their unique characteristics, such as remarkable stability, functional versatility, and precise regulatory capabilities, make them a promising class of molecules for personalized cancer treatment. Nevertheless, the field remains in its early stages, and inconsistencies in studies, along with technical limitations, underscore the urgent requirement for rigorous and standardized research frameworks. The future clinical impact of circRNAs is dependent on ongoing methodological advancements, scalable delivery systems, and robust clinical validation. As research into circRNAs continues to gain momentum, several remarkable methods and techniques are emerging that could reshape malignancy diagnosis and treatment. The advancement of CRISPR-based technologies and the development of chemically stabilized synthetic circRNAs may improve specificity and durability. Alongside, progress in nanoparticle and exosome-mediated delivery platform provides more targeted and efficient delivery of circRNA-based therapeutics, increasing their clinical potential.^185^ Regardless of these developments, important challenges remain, such as lack of clinical trial data, functional diversity of circRNAs across various cancer types, and an incomplete understanding of circRNA-host gene interactions. Future studies efforts should prioritize large-scale, standardized investigations accompanied by robust functional validation across a variety ofpreclinical and clinical models. Also, integrating circRNA signatures with genomic and proteomic profiling could facilitate highly personalized cancer management, paving the way for accurate oncology tools that using circRNA biology for personalized prognosis, diagnosis, and treatment interventions.^81,186^

## Competing Interests

 The authors declare that they have no known competing financial interests or personal relationships that could have appeared to influence the work reported in this paper.

## Consent for Publication

 Not applicable.

## Data Availability Statement

 Not applicable.

## Ethical Approval

 Not applicable.

## References

[1] Beňačka R, Szabóová D, Guľašová Z, Hertelyová Z, Radoňak J (2023). Non-coding RNAs in human cancer and other diseases: overview of the diagnostic potential. Int J Mol Sci.

[2] Aftabi Y, Ansarin K, Shanehbandi D, Khalili M, Seyedrezazadeh E, Rahbarnia L (2021). Long non-coding RNAs as potential biomarkers in the prognosis and diagnosis of lung cancer: a review and target analysis. IUBMB Life.

[3] Mansoori B, Sandoghchian Shotorbani S, Baradaran B (2014). RNA interference and its role in cancer therapy. Adv Pharm Bull.

[4] Esteller M (2011). Non-coding RNAs in human disease. Nat Rev Genet.

[5] Shanehbandi D, Asadi M, Seyedrezazadeh E, Zafari V, Shekari N, Akbari M (2023). MicroRNA-based biomarkers in lung cancer: recent advances and potential applications. Curr Mol Med.

[6] Kamran S, Seyedrezazadeh E, Shanehbandi D, Asadi M, Zafari V, Shekari N (2022). Combination therapy with KRAS and P38α siRNA suppresses colorectal cancer growth and development in SW480 cell line. J Gastrointest Cancer.

[7] Mercer TR, Dinger ME, Mattick JS (2009). Long non-coding RNAs: insights into functions. Nat Rev Genet.

[8] Romano G, Veneziano D, Acunzo M, Croce CM (2017). Small non-coding RNA and cancer. Carcinogenesis.

[9] Vo JN, Cieslik M, Zhang Y, Shukla S, Xiao L, Zhang Y, et al. The landscape of circular RNA in cancer. Cell 2019;176(4):869-81.e13. doi: 10.1016/j.cell.2018.12.021. PMC6601354 30735636

[10] Will CL, Lührmann R (2011). Spliceosome structure and function. Cold Spring HarbPerspect Biol.

[11] Ivanov A, Memczak S, Wyler E, Torti F, Porath HT, Orejuela MR (2015). Analysis of intron sequences reveals hallmarks of circular RNA biogenesis in animals. Cell Rep.

[12] Wang J, Wang L (2019). Deep learning of the back-splicing code for circular RNA formation. Bioinformatics.

[13] Xiao MS, Ai Y, Wilusz JE (2020). Biogenesis and functions of circular RNAs come into focus. Trends Cell Biol.

[14] Zhang XO, Dong R, Zhang Y, Zhang JL, Luo Z, Zhang J (2016). Diverse alternative back-splicing and alternative splicing landscape of circular RNAs. Genome Res.

[15] Ebbesen KK, Hansen TB, Kjems J (2017). Insights into circular RNA biology. RNA Biol.

[16] Hsiao KY, Sun HS, Tsai SJ (2017). Circular RNA - new member of noncoding RNA with novel functions. Exp Biol Med (Maywood).

[17] Taheri M, Najafi S, Basiri A, Hussen BM, Baniahmad A, Jamali E (2021). The role and clinical potentials of circular RNAs in prostate cancer. Front Oncol.

[18] Zeng K, Chen X, Xu M, Liu X, Hu X, Xu T (2018). CircHIPK3 promotes colorectal cancer growth and metastasis by sponging miR-7. Cell Death Dis.

[19] Han D, Li J, Wang H, Su X, Hou J, Gu Y (2017). Circular RNA circMTO1 acts as the sponge of microRNA-9 to suppress hepatocellular carcinoma progression. Hepatology.

[20] Araghi M, Soerjomataram I, Jenkins M, Brierley J, Morris E, Bray F (2019). Global trends in colorectal cancer mortality: projections to the year 2035. Int J Cancer.

[21] Lasda E, Parker R (2014). Circular RNAs: diversity of form and function. RNA.

[22] Jeck WR, Sharpless NE (2014). Detecting and characterizing circular RNAs. Nat Biotechnol.

[23] Chen I, Chen CY, Chuang TJ (2015). Biogenesis, identification, and function of exonic circular RNAs. Wiley Interdiscip Rev RNA.

[24] Greene J, Baird AM, Brady L, Lim M, Gray SG, McDermott R (2017). Circular RNAs: biogenesis, function and role in human diseases. Front Mol Biosci.

[25] Ebbesen KK, Kjems J, Hansen TB (2016). Circular RNAs: identification, biogenesis and function. BiochimBiophys Acta.

[26] Patop IL, Wüst S, Kadener S (2019). Past, present, and future of circRNAs. EMBO J.

[27] Li M, Ding W, Sun T, Tariq MA, Xu T, Li P (2018). Biogenesis of circular RNAs and their roles in cardiovascular development and pathology. FEBS J.

[28] Pandey PR, Rout PK, Das A, Gorospe M, Panda AC (2019). RPAD (RNase R treatment, polyadenylation, and poly(A) + RNA depletion) method to isolate highly pure circular RNA. Methods.

[29] Ashwal-Fluss R, Meyer M, Pamudurti NR, Ivanov A, Bartok O, Hanan M (2014). circRNA biogenesis competes with pre-mRNA splicing. Mol Cell.

[30] Cao D (2021). Reverse complementary matches simultaneously promote both back-splicing and exon-skipping. BMC Genomics.

[31] Daniel C, Behm M, Öhman M (2015). The role of Alu elements in the cis-regulation of RNA processing. Cell Mol Life Sci.

[32] Barrett SP, Wang PL, Salzman J (2015). Circular RNA biogenesis can proceed through an exon-containing lariat precursor. Elife.

[33] Bachvaroff TR, Place AR (2008). From stop to start: tandem gene arrangement, copy number and trans-splicing sites in the dinoflagellate Amphidiniumcarterae. PLoS One.

[34] Aravin AA, Naumova NM, Tulin AV, Vagin VV, Rozovsky YM, Gvozdev VA (2001). Double-stranded RNA-mediated silencing of genomic tandem repeats and transposable elements in the D melanogaster germline. Curr Biol.

[35] Shi Y, Fang N, Li Y, Guo Z, Jiang W, He Y (2020). Circular RNA LPAR3 sponges microRNA-198 to facilitate esophageal cancer migration, invasion, and metastasis. Cancer Sci.

[36] Yin Y, Long J, He Q, Li Y, Liao Y, He P (2019). Emerging roles of circRNA in formation and progression of cancer. J Cancer.

[37] Yao T, Chen Q, Fu L, Guo J (2017). Circular RNAs: biogenesis, properties, roles, and their relationships with liver diseases. Hepatol Res.

[38] Chen LL, Yang L (2015). Regulation of circRNA biogenesis. RNA Biol.

[39] Ragan C, Goodall GJ, Shirokikh NE, Preiss T (2019). Insights into the biogenesis and potential functions of exonic circular RNA. Sci Rep.

[40] Vidal AF (2020). Read-through circular RNAs reveal the plasticity of RNA processing mechanisms in human cells. RNA Biol.

[41] Guarnerio J, Bezzi M, Jeong JC, Paffenholz SV, Berry K, Naldini MM (2016). Oncogenic role of fusion-circRNAs derived from cancer-associated chromosomal translocations. Cell.

[42] Wu X, Tao P, Zhou Q, Li J, Yu Z, Wang X (2017). IL-6 secreted by cancer-associated fibroblasts promotes epithelial-mesenchymal transition and metastasis of gastric cancer via JAK2/STAT3 signaling pathway. Oncotarget.

[43] Dori M, Bicciato S (2019). Integration of bioinformatic predictions and experimental data to identify circRNA-miRNA associations. Genes (Basel).

[44] Wen Y, Li B, He M, Teng S, Sun Y, Wang G (2021). circHIPK3 promotes proliferation and migration and invasion via regulation of miR-637/HDAC4 signaling in osteosarcoma cells. Oncol Rep.

[45] Liang HF, Zhang XZ, Liu BG, Jia GT, Li WL (2017). Circular RNA circ-ABCB10 promotes breast cancer proliferation and progression through sponging miR-1271. Am J Cancer Res.

[46] Hansen TB, Jensen TI, Clausen BH, Bramsen JB, Finsen B, Damgaard CK (2013). Natural RNA circles function as efficient microRNA sponges. Nature.

[47] Gu A, Jaijyan DK, Yang S, Zeng M, Pei S, Zhu H (2023). Functions of circular RNA in human diseases and illnesses. Noncoding RNA.

[48] Du WW, Yang W, Chen Y, Wu ZK, Foster FS, Yang Z (2017). Foxo3 circular RNA promotes cardiac senescence by modulating multiple factors associated with stress and senescence responses. Eur Heart J.

[49] Hsiao KY, Lin YC, Gupta SK, Chang N, Yen L, Sun HS (2017). Noncoding effects of circular RNA CCDC66 promote colon cancer growth and metastasis. Cancer Res.

[50] Wu J, Jiang Z, Chen C, Hu Q, Fu Z, Chen J (2018). CircIRAK3 sponges miR-3607 to facilitate breast cancer metastasis. Cancer Lett.

[51] Luan W, Shi Y, Zhou Z, Xia Y, Wang J (2018). circRNA_0084043 promote malignant melanoma progression via miR-153-3p/Snail axis. BiochemBiophys Res Commun.

[52] Zeng K, He B, Yang BB, Xu T, Chen X, Xu M (2018). The pro-metastasis effect of circANKS1B in breast cancer. Mol Cancer.

[53] Liu Y, Lu C, Zhou Y, Zhang Z, Sun L (2018). Circular RNA hsa_circ_0008039 promotes breast cancer cell proliferation and migration by regulating miR-432-5p/E2F3 axis. BiochemBiophys Res Commun.

[54] Wang H, Xiao Y, Wu L, Ma D (2018). Comprehensive circular RNA profiling reveals the regulatory role of the circRNA-000911/miR-449a pathway in breast carcinogenesis. Int J Oncol.

[55] Zhong Q, Huang J, Wei J, Wu R (2019). Circular RNA CDR1as sponges miR-7-5p to enhance E2F3 stability and promote the growth of nasopharyngeal carcinoma. Cancer Cell Int.

[56] Hansen TB, Kjems J, Damgaard CK (2013). Circular RNA and miR-7 in cancer. Cancer Res.

[57] Yao Z, Luo J, Hu K, Lin J, Huang H, Wang Q (2017). ZKSCAN1 gene and its related circular RNA (circZKSCAN1) both inhibit hepatocellular carcinoma cell growth, migration, and invasion but through different signaling pathways. Mol Oncol.

[58] Lei B, Tian Z, Fan W, Ni B (2019). Circular RNA: a novel biomarker and therapeutic target for human cancers. Int J Med Sci.

[59] Huang XY, Huang ZL, Xu YH, Zheng Q, Chen Z, Song W (2017). Comprehensive circular RNA profiling reveals the regulatory role of the circRNA-100338/miR-141-3p pathway in hepatitis B-related hepatocellular carcinoma. Sci Rep.

[60] Han C, Seebacher NA, Hornicek FJ, Kan Q, Duan Z (2017). Regulation of microRNAs function by circular RNAs in human cancer. Oncotarget.

[61] Wan L, Zhang L, Fan K, Cheng ZX, Sun QC, Wang JJ (2016). Circular RNA-ITCH suppresses lung cancer proliferation via inhibiting the Wnt/β-catenin pathway. Biomed Res Int.

[62] Cong L, Yang Q, Hu C, Yu Q, Hao S, Li D (2019). Current status of functional studies on circular RNAs in bladder cancer and their potential role as diagnostic and prognostic biomarkers: a review. Med Sci Monit.

[63] Wang L, Xu C, Wang C, Gong W, Zhang K, Chen Q (2018). Circ-PAX2 promotes proliferation and metastasis by absorbing miR-186 in lung cancer cells. Int J Clin Exp Pathol.

[64] Lizarbe MA, Calle-Espinosa J, Fernández-Lizarbe E, Fernández-Lizarbe S, Robles M, Olmo N (2017). Colorectal cancer: from the genetic model to posttranscriptional regulation by noncoding RNAs. Biomed Res Int.

[65] Lu J, Zhu Y, Qin Y, Chen Y (2020). CircNFIX acts as a miR-212-3p sponge to enhance the malignant progression of non-small cell lung cancer by up-regulating ADAM10. Cancer Manag Res.

[66] Xie H, Ren X, Xin S, Lan X, Lu G, Lin Y (2016). Emerging roles of circRNA_001569 targeting miR-145 in the proliferation and invasion of colorectal cancer. Oncotarget.

[67] Wang HY, Wang YP, Zeng X, Zheng Y, Guo QH, Ji R (2020). Circular RNA is a popular molecule in tumors of the digestive system (Review). Int J Oncol.

[68] Kun-Peng Z, Xiao-Long M, Chun-Lin Z (2018). Overexpressed circPVT1, a potential new circular RNA biomarker, contributes to doxorubicin and cisplatin resistance of osteosarcoma cells by regulating ABCB1. Int J Biol Sci.

[69] Zhang J, Liu H, Hou L, Wang G, Zhang R, Huang Y (2017). Circular RNA_LARP4 inhibits cell proliferation and invasion of gastric cancer by sponging miR-424-5p and regulating LATS1 expression. Mol Cancer.

[70] Chen G, Shi Y, Liu M, Sun J (2018). circHIPK3 regulates cell proliferation and migration by sponging miR-124 and regulating AQP3 expression in hepatocellular carcinoma. Cell Death Dis.

[71] Xia W, Qiu M, Chen R, Wang S, Leng X, Wang J (2016). Circular RNA has_circ_0067934 is upregulated in esophageal squamous cell carcinoma and promoted proliferation. Sci Rep.

[72] Qu S, Liu Z, Yang X, Zhou J, Yu H, Zhang R (2018). The emerging functions and roles of circular RNAs in cancer. Cancer Lett.

[73] Lu WY (2017). Roles of the circular RNA circ-Foxo3 in breast cancer progression. Cell Cycle.

[74] Du WW, Fang L, Yang W, Wu N, Awan FM, Yang Z (2017). Induction of tumor apoptosis through a circular RNA enhancing Foxo3 activity. Cell Death Differ.

[75] Rong MH, Li JD, Zhong LY, Huang YZ, Chen J, Xie LY (2022). CCNB1 promotes the development of hepatocellular carcinoma by mediating DNA replication in the cell cycle. Exp Biol Med (Maywood).

[76] Pamudurti NR, Konakondla-Jacob VV, Krishnamoorthy A, Ashwal-Fluss R, Bartok O, Wüst S, et al. An in vivo knockdown strategy reveals multiple functions for circMbl. bioRxiv [Preprint]. November 29, 2018. Available from: https://www.biorxiv.org/content/10.1101/483271v1.

[77] Konakondla JV. Molecular Interaction of circMbl and MBL in Vivo [dissertation]. Brandeis University Graduate School of Arts and Sciences; 2019.

[78] Li XX, Xiao L, Chung HK, Ma XX, Liu X, Song JL (2020). Interaction between HuR and circPABPN1 modulates autophagy in the intestinal epithelium by altering ATG16L1 translation. Mol Cell Biol.

[79] Abdelmohsen K, Panda AC, Munk R, Grammatikakis I, Dudekula DB, De S (2017). Identification of HuR target circular RNAs uncovers suppression of PABPN1 translation by CircPABPN1. RNA Biol.

[80] Liu B, Tian Y, Chen M, Shen H, Xia J, Nan J (2021). CircUBAP2 promotes MMP9-mediated oncogenic effect via sponging miR-194-3p in hepatocellular carcinoma. Front Cell Dev Biol.

[81] Kundu I, Varshney S, Karnati S, Naidu S (2024). The multifaceted roles of circular RNAs in cancer hallmarks: from mechanisms to clinical implications. Mol Ther Nucleic Acids.

[82] Li Z, Huang C, Bao C, Chen L, Lin M, Wang X (2015). Exon-intron circular RNAs regulate transcription in the nucleus. Nat Struct Mol Biol.

[83] Hu Q, Zhou T (2018). EIciRNA-mediated gene expression: tunability and bimodality. FEBS Lett.

[84] Bose R, Ain R (2018). Regulation of transcription by circular RNAs. Adv Exp Med Biol.

[85] Lu D, Xu AD (2016). Mini review: circular RNAs as potential clinical biomarkers for disorders in the central nervous system. Front Genet.

[86] Zlotorynski E (2018). Splicing: Going in circles. Nat Rev Genet.

[87] Yang Z, He T, Chen Q (2021). The Roles of CircRNAs in Regulating Muscle Development of Livestock Animals. Front Cell Dev Biol.

[88] Zhang J, Chen Z, Liu X, Yang C, Xie D (2021). Gain of circBRAF Represses Glioma Progression by Regulating miR-1290/FBXW7 Axis. Neurochemical research.

[89] Shao T, Pan YH, Xiong XD (2021). Circular RNA: an important player with multiple facets to regulate its parental gene expression. Mol Ther Nucleic Acids.

[90] Granados-Riveron JT, Aquino-Jarquin G (2016). The complexity of the translation ability of circRNAs. BiochimBiophys Acta.

[91] Legnini I, Di Timoteo G, Rossi F, Morlando M, Briganti F, Sthandier O, et al. Circ-ZNF609 is a circular RNA that can be translated and functions in myogenesis. Mol Cell 2017;66(1):22-37.e9. doi: 10.1016/j.molcel.2017.02.017. PMC5387670 28344082

[92] Zhang C, Ma L, Niu Y, Wang Z, Xu X, Li Y (2020). Circular RNA in lung cancer research: biogenesis, functions, and roles. Int J Biol Sci.

[93] Koch L (2017). RNA: translated circular RNAs. Nat Rev Genet.

[94] Li I, Chen YG (2021). Emerging roles of circular RNAs in innate immunity. CurrOpin Immunol.

[95] Yu T, Wang Y, Fan Y, Fang N, Wang T, Xu T (2019). circRNAs in cancer metabolism: a review. J Hematol Oncol.

[96] Bhat AA, Gupta G, Dahiya R, Thapa R, Gahtori A, Shahwan M (2024). circRNAs: pivotal modulators of TGF-β signalling in cancer pathogenesis. Noncoding RNA Res.

[97] Pandey PR, Munk R, Kundu G, De S, Abdelmohsen K, Gorospe M (2020). Methods for analysis of circular RNAs. Wiley Interdiscip Rev RNA.

[98] Gao Y, Zhao F (2018). Computational strategies for exploring circular RNAs. Trends Genet.

[99] Zhang J, Hou L, Zuo Z, Ji P, Zhang X, Xue Y (2021). Comprehensive profiling of circular RNAs with nanopore sequencing and CIRI-long. Nat Biotechnol.

[100] Li S, Teng S, Xu J, Su G, Zhang Y, Zhao J (2019). Microarray is an efficient tool for circRNA profiling. Brief Bioinform.

[101] Szabo L, Salzman J (2016). Detecting circular RNAs: bioinformatic and experimental challenges. Nat Rev Genet.

[102] Schneider T, Schreiner S, Preußer C, Bindereif A, Rossbach O (2018). Northern blot analysis of circular RNAs. Methods Mol Biol.

[103] Panda AC, Gorospe M (2018). Detection and analysis of circular RNAs by RT-PCR. Bio Protoc.

[104] Li T, Shao Y, Fu L, Xie Y, Zhu L, Sun W (2018). Plasma circular RNA profiling of patients with gastric cancer and their droplet digital RT-PCR detection. J Mol Med (Berl).

[105] Chen DF, Zhang LJ, Tan K, Jing Q (2018). Application of droplet digital PCR in quantitative detection of the cell-free circulating circRNAs. BiotechnolBiotechnol Equip.

[106] Zirkel A, Papantonis A (2018). Detecting circular RNAs by RNA fluorescence in situ hybridization. Methods Mol Biol.

[107] Dudekula DB, Panda AC, Grammatikakis I, De S, Abdelmohsen K, Gorospe M (2016). CircInteractome: a web tool for exploring circular RNAs and their interacting proteins and microRNAs. RNA Biol.

[108] Suzuki H, Tsukahara T (2014). A view of pre-mRNA splicing from RNase R resistant RNAs. Int J Mol Sci.

[109] Xie R, Zhang Y, Zhang J, Li J, Zhou X (2020). The role of circular RNAs in immune-related diseases. Front Immunol.

[110] Xiao Y, Qiu M, Tan C, Huang W, Hu S, Jiang X (2022). Systematic analysis of circRNA biomarkers for diagnosis, prognosis and therapy in colorectal cancer. Front Genet.

[111] Gao L, Fan J, He J, Fan W, Che X, Wang X (2024). Circular RNA as diagnostic and prognostic biomarkers in hematological malignancies: systematic review. Technol Cancer Res Treat.

[112] Wang M, Xie F, Lin J, Zhao Y, Zhang Q, Liao Z (2021). Diagnostic and prognostic value of circulating circRNAs in cancer. Front Med (Lausanne).

[113] Yang Q, Li F, He AT, Yang BB (2021). Circular RNAs: expression, localization, and therapeutic potentials. Mol Ther.

[114] Xu C, Jun E, Okugawa Y, Toiyama Y, Borazanci E, Bolton J, et al. A circulating panel of circRNA biomarkers for the noninvasive and early detection of pancreatic ductal adenocarcinoma. Gastroenterology 2024;166(1):178-90.e16. doi: 10.1053/j.gastro.2023.09.050. PMC10843014 37839499

[115] Qin M, Liu G, Huo X, Tao X, Sun X, Ge Z (2016). Hsa_circ_0001649: a circular RNA and potential novel biomarker for hepatocellular carcinoma. Cancer Biomark.

[116] Zhang ZC, Guo XL, Li X (2018). The novel roles of circular RNAs in metabolic organs. Genes Dis.

[117] Pan Y, Mao Y, Jin R, Jiang L (2018). Crosstalk between the Notch signaling pathway and non-coding RNAs in gastrointestinal cancers. Oncol Lett.

[118] Chen X, Chen RX, Wei WS, Li YH, Feng ZH, Tan L (2018). PRMT5 circular RNA promotes metastasis of urothelial carcinoma of the bladder through sponging miR-30c to induce epithelial-mesenchymal transition. Clin Cancer Res.

[119] Yu CY, Kuo HC (2019). The emerging roles and functions of circular RNAs and their generation. J Biomed Sci.

[120] Zhao SY, Wang J, Ouyang SB, Huang ZK, Liao L (2018). Salivary circular RNAs hsa_circ_0001874 and hsa_circ_0001971 as novel biomarkers for the diagnosis of oral squamous cell carcinoma. Cell PhysiolBiochem.

[121] Yin WB, Yan MG, Fang X, Guo JJ, Xiong W, Zhang RP (2018). Circulating circular RNA hsa_circ_0001785 acts as a diagnostic biomarker for breast cancer detection. Clin Chim Acta.

[122] Gong Y, Mao J, Wu D, Wang X, Li L, Zhu L (2018). Circ-ZEB133 promotes the proliferation of human HCC by sponging miR-200a-3p and upregulating CDK6. Cancer Cell Int.

[123] Shang X, Li G, Liu H, Li T, Liu J, Zhao Q (2016). Comprehensive circular RNA profiling reveals that hsa_circ_0005075, a new circular RNA biomarker, is involved in hepatocellular crcinoma development. Medicine (Baltimore).

[124] Xuan L, Qu L, Zhou H, Wang P, Yu H, Wu T (2016). Circular RNA: a novel biomarker for progressive laryngeal cancer. Am J Transl Res.

[125] Xu ZQ, Yang MG, Liu HJ, Su CQ (2018). Circular RNA hsa_circ_0003221 (circPTK2) promotes the proliferation and migration of bladder cancer cells. J Cell Biochem.

[126] Wang X, Zhang Y, Huang L, Zhang J, Pan F, Li B (2015). Decreased expression of hsa_circ_001988 in colorectal cancer and its clinical significances. Int J Clin Exp Pathol.

[127] Li P, Chen S, Chen H, Mo X, Li T, Shao Y (2015). Using circular RNA as a novel type of biomarker in the screening of gastric cancer. Clin Chim Acta.

[128] Dong Y, Gao Q, Chen Y, Zhang Z, Du Y, Liu Y (2023). Identification of circRNA signature associated with tumor immune infiltration to predict therapeutic efficacy of immunotherapy. Nat Commun.

[129] Li LX, Hao Y, Dong L, Qiao ZQ, Yang SC, Chen YD (2025). Circular RNAs as biomarkers in breast cancer diagnosis, prognosis, molecular types, metastasis and drug resistance. Technol Cancer Res Treat.

[130] Wu X, Shi M, Lian Y, Zhang H (2023). Exosomal circRNAs as promising liquid biopsy biomarkers for glioma. Front Immunol.

[131] Yi J, Wang L, Hu GS, Zhang YY, Du J, Ding JC (2023). CircPVT1 promotes ER-positive breast tumorigenesis and drug resistance by targeting ESR1 and MAVS. EMBO J.

[132] Tierno D, Grassi G, Zanconati F, Dapas B, Scaggiante B (2024). Plasma circular RNAs as biomarkers for breast cancer. Biomedicines.

[133] Sarkar D, Diermeier SD (2021). Circular RNAs: potential applications as therapeutic targets and biomarkers in breast cancer. Noncoding RNA.

[134] Yan Y, Su M, Qin B (2020). CircHIPK3 promotes colorectal cancer cells proliferation and metastasis via modulating of miR-1207-5p/FMNL2 signal. BiochemBiophys Res Commun.

[135] Li Y, Zheng F, Xiao X, Xie F, Tao D, Huang C (2017). CircHIPK3 sponges miR-558 to suppress heparanase expression in bladder cancer cells. EMBO Rep.

[136] Li F, Zhang L, Li W, Deng J, Zheng J, An M (2015). Circular RNA ITCH has inhibitory effect on ESCC by suppressing the Wnt/β-catenin pathway. Oncotarget.

[137] Su Y, Lv X, Yin W, Zhou L, Hu Y, Zhou A (2019). circRNA Cdr1as functions as a competitive endogenous RNA to promote hepatocellular carcinoma progression. Aging (Albany NY).

[138] Sang M, Meng L, Liu S, Ding P, Chang S, Ju Y (2018). Circular RNA ciRS-7 maintains metastatic phenotypes as a ceRNA of miR-1299 to target MMPs. Mol Cancer Res.

[139] Xie X, Sun FK, Huang X, Wang CH, Dai J, Zhao JP (2021). A circular RNA, circSMARCA5, inhibits prostate cancer proliferative, migrative, and invasive capabilities via the miR-181b-5p/miR-17-3p-TIMP3 axis. Aging (Albany NY).

[140] Yang Y, Gao X, Zhang M, Yan S, Sun C, Xiao F (2018). Novel role of FBXW7 circular RNA in repressing glioma tumorigenesis. J Natl Cancer Inst.

[141] Zhang Y, Zhao H, Zhang L (2018). Identification of the tumor-suppressive function of circular RNA FOXO3 in non-small cell lung cancer through sponging miR-155. Mol Med Rep.

[142] Kong Z, Wan X, Lu Y, Zhang Y, Huang Y, Xu Y (2020). Circular RNA circFOXO3 promotes prostate cancer progression through sponging miR-29a-3p. J Cell Mol Med.

[143] Fang G, Ye BL, Hu BR, Ruan XJ, Shi YX (2018). circRNA_100290 promotes colorectal cancer progression through miR-516b-induced downregulation of FZD4 expression and Wnt/β-catenin signaling. BiochemBiophys Res Commun.

[144] Li H, Xue S, Zhang X, Li F, Bei S, Feng L (2022). circRNA PVT1 modulated cell migration and invasion through Epithelial-Mesenchymal Transition (EMT) mediation in gastric cancer through miR-423-5p/Smad3 pathway. Regen Ther.

[145] Liu YP, Wan J, Long F, Tian J, Zhang C (2020). circPVT1 facilitates invasion and metastasis by regulating miR-205-5p/c-FLIP axis in osteosarcoma. Cancer Manag Res.

[146] Sang Y, Chen B, Song X, Li Y, Liang Y, Han D (2019). circRNA_0025202 regulates tamoxifen sensitivity and tumor progression via regulating the miR-182-5p/FOXO3a axis in breast cancer. Mol Ther.

[147] He R, Liu P, Xie X, Zhou Y, Liao Q, Xiong W (2017). circGFRA1 and GFRA1 act as ceRNAs in triple negative breast cancer by regulating miR-34a. J Exp Clin Cancer Res.

[148] Zhang PF, Gao C, Huang XY, Lu JC, Guo XJ, Shi GM (2020). Cancer cell-derived exosomal circUHRF1 induces natural killer cell exhaustion and may cause resistance to anti-PD1 therapy in hepatocellular carcinoma. Mol Cancer.

[149] Qiu M, Xia W, Chen R, Wang S, Xu Y, Ma Z (2018). The circular RNA circPRKCI promotes tumor growth in lung adenocarcinoma. Cancer Res.

[150] Wang S, Xue X, Wang R, Li X, Li Q, Wang Y (2018). CircZNF609 promotes breast cancer cell growth, migration, and invasion by elevating p70S6K1 via sponging miR-145-5p. Cancer Manag Res.

[151] Rinaldi C, Wood MJA (2018). Antisense oligonucleotides: the next frontier for treatment of neurological disorders. Nat Rev Neurol.

[152] Cao SQ, Xue ST, Li WJ, Hu GS, Wu ZG, Zheng JC (2024). CircHIPK3 regulates fatty acid metabolism through miR-637/FASN axis to promote esophageal squamous cell carcinoma. Cell Death Discov.

[153] Pollak AJ, Zhao L, Crooke ST (2023). Systematic analysis of chemical modifications of phosphorothioate antisense oligonucleotides that modulate their innate immune response. Nucleic Acid Ther.

[154] Katti A, Diaz BJ, Caragine CM, Sanjana NE, Dow LE (2022). CRISPR in cancer biology and therapy. Nat Rev Cancer.

[155] Zhang J, Luo Z, Zheng Y, Duan M, Qiu Z, Huang C (2024). circRNA as an Achilles heel of cancer: characterization, biomarker and therapeutic modalities. J Transl Med.

[156] Wen L, Zhao C, Song J, Ma L, Ruan J, Xia X (2020). CRISPR/Cas9-mediated TERT disruption in cancer cells. Int J Mol Sci.

[157] Zhang Y, Nguyen TM, Zhang XO, Wang L, Phan T, Clohessy JG (2021). Optimized RNA-targeting CRISPR/Cas13d technology outperforms shRNA in identifying functional circRNAs. Genome Biol.

[158] Zhang M, Guo R, Yuan Z, Wang H (2025). Lipid nanoparticle (LNP)-a vector suitable for evolving therapies for advanced hepatocellular carcinoma (HCC). Glob Chall.

[159] Yao Y, Zhou Y, Liu L, Xu Y, Chen Q, Wang Y (2020). Nanoparticle-based drug delivery in cancer therapy and its role in overcoming drug resistance. Front Mol Biosci.

[160] Cai J, Liu Z, Chen S, Zhang J, Li H, Wang X (2025). Engineered circular RNA-based DLL3-targeted CAR-T therapy for small cell lung cancer. Exp Hematol Oncol.

[161] Ning H, Jiang Y, Li B, Ren J, Ran A, Li W (2025). Targeted delivery of circPDHK1 siRNA via aptamer functionalized lipid nanoparticles inhibits ccRCC growth and migration. Int J Pharm.

[162] You S, Luo Z, Cheng N, Wu M, Lai Y, Wang F (2023). Magnetically responsive nanoplatform targeting circRNA circ_0058051 inhibits hepatocellular carcinoma progression. Drug DelivTransl Res.

[163] Shu G, Lu X, Pan Y, Cen J, Huang K, Zhou M (2023). Exosomal circSPIRE1 mediates glycosylation of E-cadherin to suppress metastasis of renal cell carcinoma. Oncogene.

[164] Gopikrishnan M, R HC, R G, Ashour HM, Pintus G, Hammad M (2023). Therapeutic and diagnostic applications of exosomal circRNAs in breast cancer. FunctIntegr Genomics.

[165] Motoyama K, Inoue H, Mimori K, Tanaka F, Kojima K, Uetake H (2010). Clinicopathological and prognostic significance of PDCD4 and microRNA-21 in human gastric cancer. Int J Oncol.

[166] Müller S, Wedler A, Breuer J, Glaß M, Bley N, Lederer M (2020). Synthetic circular miR-21 RNA decoys enhance tumor suppressor expression and impair tumor growth in mice. NAR Cancer.

[167] Yang C, Yuan W, Yang X, Li P, Wang J, Han J (2018). Circular RNA circ-ITCH inhibits bladder cancer progression by sponging miR-17/miR-224 and regulating p21, PTEN expression. Mol Cancer.

[168] Ameli-Mojarad M, Ameli-Mojarad M, Hadizadeh M, Young C, Babini H, Nazemalhosseini-Mojarad E (2021). The effective function of circular RNA in colorectal cancer. Cancer Cell Int.

[169] Huang G, Zhu H, Shi Y, Wu W, Cai H, Chen X (2015). cir-ITCH plays an inhibitory role in colorectal cancer by regulating the Wnt/β-catenin pathway. PLoS One.

[170] Peng Y, Wang HH (2020). cir-ITCH inhibits gastric cancer migration, invasion and proliferation by regulating the Wnt/β-catenin pathway. Sci Rep.

[171] Yuan Z, Huang S, Jin X, Li S (2024). Circular RNAs in cardiovascular diseases: molecular mechanisms, therapeutic advances, and innovations. Genes (Basel).

[172] Barbagallo D, Caponnetto A, Cirnigliaro M, Brex D, Barbagallo C, D’Angeli F (2018). CircSMARCA5 inhibits migration of glioblastoma multiforme cells by regulating a molecular axis involving splicing factors SRSF1/SRSF3/PTB. Int J Mol Sci.

[173] Du WW, Yang W, Liu E, Yang Z, Dhaliwal P, Yang BB (2016). Foxo3 circular RNA retards cell cycle progression via forming ternary complexes with p21 and CDK2. Nucleic Acids Res.

[174] Meng L, Wu H, Wu J, Ding P, He J, Sang M (2024). Mechanisms of immune checkpoint inhibitors: insights into the regulation of circular RNAS involved in cancer hallmarks. Cell Death Dis.

[175] Hong W, Xue M, Jiang J, Zhang Y, Gao X (2020). Circular RNA circ-CPA4/ let-7 miRNA/PD-L1 axis regulates cell growth, stemness, drug resistance and immune evasion in non-small cell lung cancer (NSCLC). J Exp Clin Cancer Res.

[176] Yang K, Zhang J, Bao C (2021). Exosomal circEIF3K from cancer-associated fibroblast promotes colorectal cancer (CRC) progression via miR-214/PD-L1 axis. BMC Cancer.

[177] Li S, Li X, Xue W, Zhang L, Yang LZ, Cao SM (2021). Screening for functional circular RNAs using the CRISPR-Cas13 system. Nat Methods.

[178] Williford JM, Wu J, Ren Y, Archang MM, Leong KW, Mao HQ (2014). Recent advances in nanoparticle-mediated siRNA delivery. Annu Rev Biomed Eng.

[179] Sani A, Cao C, Cui D (2021). Toxicity of gold nanoparticles (AuNPs): a review. BiochemBiophys Rep.

[180] Zwolsman R, Darwish YB, Kluza E, van der Meel R (2025). Engineering lipid nanoparticles for mRNA immunotherapy. Wiley Interdiscip Rev NanomedNanobiotechnol.

[181] He AT, Liu J, Li F, Yang BB (2021). Targeting circular RNAs as a therapeutic approach: current strategies and challenges. Signal Transduct Target Ther.

[182] Holdt LM, Kohlmaier A, Teupser D (2018). Circular RNAs as therapeutic agents and targets. Front Physiol.

[183] Zhang Y, Wang Y, Su X, Wang P, Lin W (2021). The value of circulating circular RNA in cancer diagnosis, monitoring, prognosis, and guiding treatment. Front Oncol.

[184] Cai J, Qiu Z, Chi-Shing Cho W, Liu Z, Chen S, Li H (2024). Synthetic circRNA therapeutics: innovations, strategies, and future horizons. MedComm (2020).

[185] Márton É, Varga A, Domoszlai D, Buglyó G, Balázs A, Penyige A (2025). Non-coding RNAs in cancer: structure, function, and clinical application. Cancers (Basel).

[186] Zhang X, Yuan Y, Wang X, Wang H, Zhang L, He J (2024). CircWHSC1 (CircNSD2): a novel circular RNA in multiple cancers. Clin Med Insights Oncol.

